# Blockade of Pannexin-1 Channels and Purinergic P2X7 Receptors Shows Protective Effects Against Cytokines-Induced Colitis of Human Colonic Mucosa

**DOI:** 10.3389/fphar.2018.00865

**Published:** 2018-08-06

**Authors:** Erica F. Diezmos, Irit Markus, D. S. Perera, Steven Gan, Li Zhang, Shaun L. Sandow, Paul P. Bertrand, Lu Liu

**Affiliations:** ^1^Department of Pharmacology, School of Medical Sciences, University of New South Wales, Sydney, NSW, Australia; ^2^Sydney Colorectal Associates, Hurstville, NSW, Australia; ^3^School of Biotechnology and Biomolecular Sciences, University of New South Wales, Sydney, NSW, Australia; ^4^Inflammation and Healing Cluster, Faculty of Science, Health, Education and Engineering, University of the Sunshine Coast, Sunshine Coast, QLD, Australia; ^5^School of Health and Biomedical Sciences, RMIT University, Bundoora, Melbourne, VIC, Australia

**Keywords:** pannexin-1, P2X7 receptor, colonic inflammation, human colitis, tissue explants

## Abstract

**Introduction:** The pannexin-1 (Panx1) channels are found in many cell types, and ATP released from these channels can act on nearby cells activating purinergic P2X7 receptors (P2X7R) which lead to inflammation. Although Panx1 and P2X7R are implicated in the process of inflammation and cell death, few studies have looked at the role they play in inflammatory bowel disease in human. Hence, the aim of the present study was to investigate the function of Panx1 and P2X7R in an *ex vivo* colitis model developed from human colonic mucosal explants.

**Materials and Methods:** Healthy human colonic mucosal strips (4 × 10 mm) were incubated in carbogenated culture medium at 37°C for 16 h. Proinflammatory cytokines TNFα and IL-1β (each 10 ng/mL) were used to induce colitis in mucosal strips, and the effects of Panx1 and P2X7R on cytokines-induced tissue damage were determined in the presence of the Panx1 channel blocker ^10^Panx1 (100 μM) and P2X7R antagonist A438079 (100 μM). The effects of ^10^Panx1 and A438079 on cytokines-enhanced epithelial permeability were also studied using Caco-2 cells.

**Results:** Histological staining showed that the mucosal strips had severe structural damage in the cytokines-only group but not in the incubation-control group (*P* < 0.01). Compared to the cytokines-only group, crypt damage was significantly decreased in groups receiving cytokines with inhibitors (^10^Panx1, A438079, or ^10^Panx1 + A438079, *P* < 0.05). The immunoreactive signals of tight junction protein zonula occludens-1 (ZO-1) were abundant in all control tissues but were significantly disrupted and lost in the cytokines-only group (*P* < 0.01). The diminished ZO-1 immunoreactivity induced by cytokines was prevented in the presence of ^10^Panx1 (*P* = 0.04). Likewise, ^10^Panx1 significantly attenuated the cytokines-evoked increase in paracellular permeability of Caco-2 cells. Although the inhibition of P2X7R activity by A438079 diminished cytokines-induced crypt damage, its effect on the maintenance of ZO-1 immunoreactivity and Caco-2 epithelial cell integrity was less evident.

**Conclusion:** The blockade of Panx1 and P2X7R reduced the inflammatory cytokines-induced crypt damage, loss of tight junctions and increase in cell permeability. Thus, Panx1 and P2X7R may have roles in causing mucosal damage, a common clinical feature of inflammatory bowel disease.

## Introduction

The pannexin family of membrane bound channels are known for their role as conduits for the release of small molecules such as ATP (Penuela et al., 2013). Panx1 is the best studied subtype of pannexins and is often associated with the purinergic P2X7R (Locovei et al., 2007). P2X7R are often found on colonic epithelium and inflammatory cells, and can form large pores that allow molecules of up to 900 kDa to pass through (Pelegrin and Surprenant, 2009; Diezmos et al., 2016). Prolonged activation of P2X7R leads to cell death and inflammation which involves positive feedback signaling (North, 2002; Locovei et al., 2007; Kurashima et al., 2012; Idzko et al., 2014; Kuhny et al., 2014). In other tissues, Panx1 is established as a mediator of ATP release into the extracellular fluid where it can act as a paracrine molecule to activate P2X7R (Pelegrin and Surprenant, 2006; Wang et al., 2013). Considering their roles in other tissues, the interplay between Panx1 channel opening and P2X7R activation is of interest in the human colon, particularly during inflammation.

Activation of Panx1 and P2X7R has been shown to lead to changes in cytokine expression which influence inflammatory processes. Earlier studies showed that IL-1β release requires both Panx1 and P2X7R (Pelegrin and Surprenant, 2006; Riteau et al., 2010). Panx1, P2X1R, P2X4R, and P2X7R have been shown to play roles in IL-2 synthesis in T cells (Woehrle et al., 2010a,b). More recent studies in astrocytes have shown that Panx1 is required for IL-6 and IL-8 release (Wei et al., 2016). IBD is characterized by changes in cytokine levels, including elevated TNFα, IL-1β, IL-6, IL-8, IL-12, and IL-17, which are common to both ulcerative colitis and Crohn’s disease (Fiocchi, 1998; Papadakis and Targan, 2000; Sanchez-Munoz et al., 2008; Strober and Fuss, 2011).

Neutrophil and lymphocyte infiltration particularly in IBD can both influence and be influenced by cytokine activity (Chin and Parkos, 2006; Xavier and Podolsky, 2007; Sakai and Kobayashi, 2015). Monocytes can migrate to the location of tissue damage to secrete IL-1β (Khor et al., 2011). The migration and infiltration of immune cells can also lead to the amplification of local immune responses via the release of reactive oxygen species from these cells which have pro-inflammatory effects (Fort et al., 1998; Xavier and Podolsky, 2007).

Colitis models are commonly used to simulate the observed characteristics of IBD such as the cytokine expression profile and cell migration. Recently a human mucosa colitis model was developed using TNFα and IL-1β (Nicotra et al., 2013). Initial studies using this human colitis model were conducted to observe the effects of drugs including prostaglandin ethanolamides, anandamide and cannabidiol, which can be protective against pro-inflammatory cytokines (Harvey et al., 2013; Nicotra et al., 2013). TNFα and IL-1β are cytokines critical in mediating inflammatory responses in many diseases and tissue injuries. It has been shown that TNFα and IL-1β are released as a result of P2X7R and Panx1 channel activation in different cells, e.g., inflammatory, neuronal, and glial cells (Hide et al., 2000; Krizaj et al., 2014; Diezmos et al., 2016; Giuliani et al., 2017). Previous work using a DSS mouse colitis model has shown that blocking Panx1 and P2X7R function can reduce the tissue damage observed (Wei et al., 2016). Since Panx1 showed different expression patterns in human intestine between control and IBD at both the gene and protein levels, and since Panx1 and P2X7R are both expressed in the mucosal layer, they may be involved in IBD pathophysiology (Diezmos et al., 2013, 2016). Thus, it is important to elucidate the functional roles of Panx1 and P2X7R in the human intestine, especially in the context of IBD. In the present study, by using a human colitis model adapted from previous studies (Harvey et al., 2013; Nicotra et al., 2013), we aimed to determine whether inhibition of Panx1 and/or P2X7R function will prevent cytokines-induced inflammation in the mucosal layer of the human colon.

## Materials and Methods

### Specimens and Colonic Mucosa Preparation

Control colon specimens were obtained from patients who underwent colectomy for colon cancer (age ranged from 33 to 70 years, median 63 years, *n* = 19, 12 males and 7 females). Colon segments (11 sigmoid, 6 ascending, and 2 transverse) were taken at a site 10–20 cm away from the tumor and transported in ice-cold Krebs-Henseleit solution directly from the operating room to the laboratory. Criteria for the exclusion of control specimens include those with inflammation or macroscopic abnormality and those with obstruction, or those from patients who had undergone radiation therapy or chemotherapy.

The mucosal layer and some submucosal tissue were dissected from the muscularis layer. Experiments were performed within 3–5 h after tissue collection from the patient. Prior to each experiment, 4 × 10 mm strips of mucosa were cut and washed in carbogenated RPMI 1640 media containing 1% FCS and 1% penicillin–streptomycin for 3 × 10 min.

This project was approved by the South Eastern Sydney Local Health District Human Research Ethics Committee, Sydney, NSW, Australia.

### *Ex Vivo* Human Colitis Model

Each mucosal strip was placed at the bottom of a 50 mL conical centrifuge tubes containing 3 mL of RPMI 1640 media with 1% FCS and 1% penicillin–streptomycin. The tube was capped with a tiny hole on the lid to allow a constant supply of carbogen to the media (Supplementary Figure 1), and the strip and media were immersed in a circulating water bath maintained at 37°C. Following a 16 h incubation, media were collected for multiplex cytokine assays and tissues were weighed and stored in Zamboni’s fixative for histological and immunohistochemical analysis.

In this study, the term “control” denotes that two mucosal strips from each specimen were immediately placed in Zamboni’s fixative after dissection, whereas “incubation-control” refers to mucosal strips that have undergone the same incubation conditions as other tissue strips but with no addition of drugs. Mucosal strips incubated with the cytokines TNFα and IL-1β (10 ng/mL for both) were used to induce colitis. Drug treatment groups included one or both of the following: 100 μM of ^10^Panx1 (Panx1 inhibitor), and 100 μM of A438079 (P2X7R antagonist), which were added concurrently with TNFα and IL-1β. Furthermore, an additional drug-only treatment group (^10^Panx1 or A438079 only) without TNFα or IL-1β incubation was also conducted. The concentrations of ^10^Panx1 and A438079 chosen were based on our preliminary tests and the literature. ^10^Panx1 at 100 μM selectively inhibits the Panx1 channel and has been used in most published studies (e.g., Pelegrin and Surprenant, 2006). While the affinity (pIC50) of A438079 to P2X7R is around 100–300 nM (Donnelly-Roberts and Jarvis, 2007), a higher concentration of A438079 has been used in many studies. For instance, in murine microglia (Bartlett et al., 2013), rat lacrimal glands (Dartt and Hodges, 2011), and HEK293 cells expressed with P2X7R (Yan et al., 2011), 100 μM of A438079 inhibited P2X7R-mediated effects induced by ATP. Lower concentrations of A438079 have been effective in some studies, but not in others, for instance, in primary hepatocytes, acetaminophen inducing necrotic cell death was prevented by pre-treatment of A438079 at 100 μM but not at 1 μM or 10 μM (Xie et al., 2013). It is possible that since there is a “surge” in ATP levels initially due to cell death, higher concentrations of antagonists are needed to block the actions mediated by high amounts of ATP.

### Histology

Hematoxylin and eosin staining was used to look at tissue histology and to analyze crypt structure. Following 16 h incubation, fixed mucosal strips were processed and embedded into paraffin. Sections were cut (4 μm) and mounted onto poly-L-lysine coated slides which were dewaxed and rehydrated in xylene and graded ethanol solutions, respectively. Slides were incubated in hematoxylin for 5 min followed by 2 min wash in water and three dips in 1% acid alcohol. Following 1 min incubation in Scott’s blue solution and a 1 min wash in water, slides were incubated in eosin for 4 min. Slides were dehydrated in ethanol and xylene, mounted with DePex solution and coverslipped.

Toluidine blue was used to stain mast cells. Slides were prepared as described above. Sections were pre-treated in 0.5 M HCl for 10 min followed by 1 h incubation in 1% aqueous toluidine blue solution. Slides were quickly rinsed in 0.5 M HCl and water. Absolute ethanol (2 × 10 dips) and xylene incubation were conducted to dehydrate the sections and prepare for DePex mounting and coverslipping.

### Immunohistochemistry

Tissues from the colitis model were sectioned and mounted on slides, dewaxed and rehydrated as described in the previous section. Slides stained for CD45 and ZO-1 were subjected to antigen retrieval with EnVision FLEX Target Retrieval Solution, Low pH (K8005 Concentrate; Dako, North Sydney, NSW, Australia). Slides were washed with phosphate buffered saline (PBS, 0.1 M, pH = 7.4), then pre-incubated in 3% hydrogen peroxide for 5 min to reduce peroxidases. Non-specific staining was blocked by goat or donkey serum (1:10) for 30 min. A secondary antibody only control, i.e., anti-rabbit IgG staining (minus ZO-1 antibody), was performed by both DAB immunohistochemistry and immunofluorescence, and no background staining was observed.

#### Bright Field

Slides analyzed for CD45 (a pan-leukocyte marker) and IBA1 (a marker for macrophages and monocytes) were processed for 3,3′-diaminobenzidine (DAB) staining. Slides were incubated with primary antibodies overnight at room temperature. Following washing in Tris-buffered saline with Tween 20 (TBS-TX, 0.1 M, pH = 7.6) for 3 × 10 min, slides were incubated in 1:200 of either anti-rabbit or anti-goat biotinylated secondary antibodies for 2 h. Antibody information is shown in Supplementary Table 1. After 1 h incubation at room temperature with avidin-biotin complex (1:200, Vector Laboratories, Burlingame, CA, United States), the slides were stained in DAB-nickel solution until a brown signal developed (Sigma, Castle Hill, NSW, Australia), and counterstained with hematoxylin (Sigma, Castle Hill, NSW, Australia) for 1 min.

#### Immunofluorescence

Slides analyzed for ZO-1 immunoreactivity (IR) were subjected to immunofluorescence. Slides were incubated with primary antibodies overnight at room temperature. After 3 × 20 min washes in TBS-TX, slides were incubated in 1:200 of fluorophore conjugated secondary antibodies for 2 h. Antibody information is shown in Supplementary Table 1. Slides were rinsed with 3 × 20 min PBS. ProLong^®^ Gold Antifade Mountant with DAPI (P-36931; Life Technologies, Mulgrave, VIC, Australia) was applied to the sections to stain for cell nuclei, followed by cover slipping.

### Multiplex Immunoassay

The levels of cytokines and chemokines secreted from mucosal strips were simultaneously measured using the magnetic bead based ProcartaPlex Mix and Match Human 8-plex immunoassay kit, which contained beads for macrophage inflammatory protein-1 alpha (MIP-1α), MIP-1β, IL-6, IL-10, IL-17A, RANTES, IFNγ, and GM-CSF (Cat no: EPX080-19032-801, San Diego, CA, United States). Undiluted media from *ex vivo* mucosal samples were run using the kit at room temperature according to the manufacturer’s instructions. In summary, 25 μL of magnetic antibody capture bead solution was added to each well of a 96-well flat bottom plate and washed with the supplied rinse buffer using a hand-held magnetic plate washer. Media samples (25 μL) and the supplied antigen standards were then added to the wells and incubated on a shaker at 500 rpm for 2 h. After washing the plate twice as previously described, 12.5 μL of detection antibody mixture was added to each well after which the plate was incubated for 30 min on a shaker at 500 rpm. The plate was washed twice followed by the addition of 25 μL of streptavidin-PE and incubation on a shaker at 500 rpm for 30 min. The plate was washed twice and 100 μL of reading buffer was added to each well. Plates were read using the MAGPIX system (Luminex Corporation, Austin, TX, United States).

### Epithelial Permeability Measurements

The human intestinal epithelial cell line Caco-2 cells (passage 45–50, originally from American Type Culture Collection, Manassas, VA, United States) were maintained in 75 cm^2^ flasks in complete DMEM medium (Sigma-Aldrich, Inc., St. Louis, MO, United States), supplemented with 10% FBS, 1000 mg/L glucose, 0.584 gm/L L-glutamine, 1% non-essential amino acids (Sigma-Aldrich, Inc., MO, St. Louis, United States) and 1% antibiotic-antimycotic solution (Gibco, Life Technologies, Inc., United States), and cultured at 37°C with 5% CO_2_. For cell permeability studies, Caco-2 cells were enzymatically detached from the flasks by trypsin EDTA and resuspended in complete DMEM medium and then plated on 24-well plates (Corning Inc., Lowell, MA, United States) containing Transwell inserts of 6.5 mm diameter and 0.4 μm membrane pore size at the density of 20,000 cells per insert. Cells were further cultured in complete DMEM medium (0.6 mL in the basolateral compartment and 0.4 mL in the apical compartment) for up to 21 days and the culture media were renewed every 2 days. The establishment of the polarized epithelial monolayers was monitored by measuring *trans*-epithelial electrical resistance (TEER) using the EVOM^2^ Epithelial Voltohmmeter (World Precision Instruments, Inc., Sarasota, FL, United States). When TEER values plateaued around 1000 Ohms above the background level, the TEER measurements were recorded at time 0, 20, and 48 h. All drugs were administered via the basolateral compartment. Cytokines, TNFα, and IL-1β (both 100 ng/mL), were applied to induce cell permeability. When the inhibitors, ^10^Panx1 (100 μM), carbenoxolone (CBX, 100μM), or A438079 (100 μM) was applied, cells were pre-treated for 15 min with the inhibitors before the addition of cytokines.

### Data Analysis

Histology and immunohistochemistry analyses were conducted using ImageJ (Wayne Rasband, National Institutes of Health, United States). Crypt damage was assessed by analyzing the percentage area damage in HE sections on two representative fields of view at 20× magnification, following the methodologies from previous studies (Ma et al., 2004; Kimball et al., 2006; Harvey et al., 2013).

The cell counting method was adapted from a previous study and was conducted at 40× magnification using tools from ImageJ (Grishagin, 2015). The following method was used to process the images for analysis: images of the mucosa and remaining submucosa were obtained, the IHC Toolbox plugin was used to remove the blue color from images, images were converted to 8-bit gray scale, a threshold was applied to highlight positive cells, and converted to mask. Artifacts from images were deleted to reduce false positive results. The images were run through the ‘Analyze Particles’ command with the following settings: size, 100-infinity pixel units^2^; circularity, 0–1. Comparison of manual versus automatic counting methods is shown in Supplementary Figure 2.

ZO-1 scoring was conducted using the criteria in **Table 1** using four fields of view at 40× magnification. The mean score was determined for each treatment group and results were expressed as mean ± SEM.

**Table 1:** Criteria used for semi-quantitative analysis of ZO-1 immunoreactivity (IR).

Score	Criteria
5	ZO-1-IR widely present on lumenal epithelium and crypts.
4	Majority of both lumen and crypt ZO-1-IR present, but some ZO-1-IR absent.
3	ZO-1-IR present on approximately 50% of lumenal epithelial cells and crypts.
3	ZO-1-IR present on majority of crypts. ZO-1-IR on lumenal epithelial cells limited or absent.
3	ZO-1-IR present on majority of lumenal epithelial cells. ZO-1-IR on crypts limited or absent.
2	Majority of both lumen and crypt ZO-1-IR absent. Some ZO-1-IR is present.
1	ZO-1-IR is fully absent from the section.


All data were analyzed using one-way ANOVA with Holm–Sidak multiple comparison tests, except for Caco-2 TEER data, for which a two-way ANOVA analysis was performed. The significance level was set at *P* < 0.05.

## Results

### Optimization of Incubation Conditions for the *ex Vivo* Model

**FIGURE 1:**
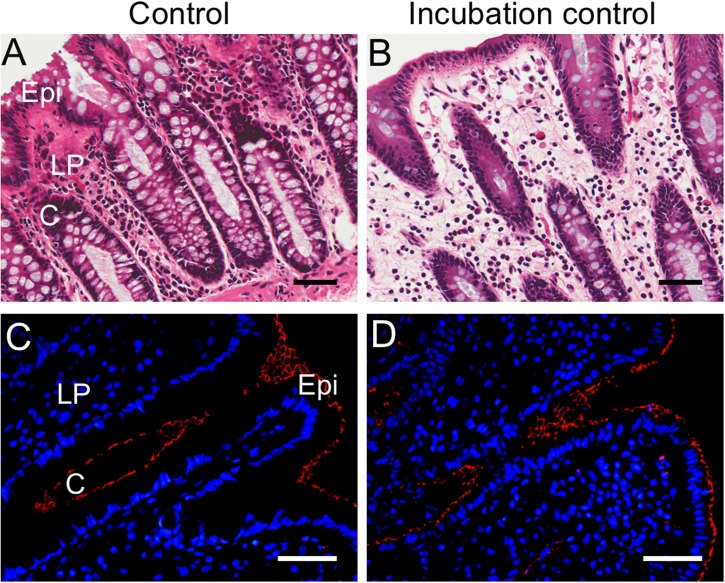
Histological comparison of control and incubation-control mucosal sections from the cytokine-induced colitis model. **(A)** Hematoxylin and eosin (HE) staining of control mucosa from tissue fixed with Zamboni’s immediately following dissection. **(B)** HE staining of incubation-control mucosa which has undergone 16 h incubation in RPMI 1640 media, 1% FCS and 1% penicillin–streptomycin before fixation in Zamboni’s. **(C)** ZO-1 immunoreactive (IR) tight junctions (red) in control mucosa fixed immediately in Zamboni’s. **(D)** ZO-1-IR tight junctions (red) in incubation-control mucosa. 4′,6-diamidino-2-phenylindole (DAPI) nuclear marker (blue). Scale bars represent 50 μm. C, crypt; Epi, epithelium; LP, lamina propria. **(A,B)** are from a 66-year-old male patient, and **(C,D)** from a 63-year-old male patient.

The conditions for the *ex vivo* colitis model were initially tested with different incubation times, between 4 and 20 h, and the results showed that mucosal integrity was maintained up to a 16 h incubation period, having similar appearance to control tissues fixed immediately in Zamboni’s fixative following dissection (Supplementary Figure 3). Lumenal and crypt structures were maintained (**Figures 1A,B**) and ZO-1-IR positive tight junctions remained intact in both control and incubation-control mucosal strips (**Figures 1C,D**). Comparison of control and incubation-control mucosal tissues in other histology experiments conducted for the present study did not show any obvious differences (Supplementary Figure 3). The percentage crypt damage (the mean ± SEM) in “incubation control” was 7.24 ± 2.48% compared to the “control” value of 2.39 ± 0.39% (n = 5, *P* = 0.09 paired *t*-test). In addition, four human colon mucosal specimens included an additional drug-only treatment (^10^Panx1 or A438079 only) without TNFα or IL-1β incubation, which did not show histological differences compared to the incubation-control group (data not shown).

### Histological Analysis of Cytokine-Induced Colitis Model

#### Crypt Damage

**FIGURE 2:**
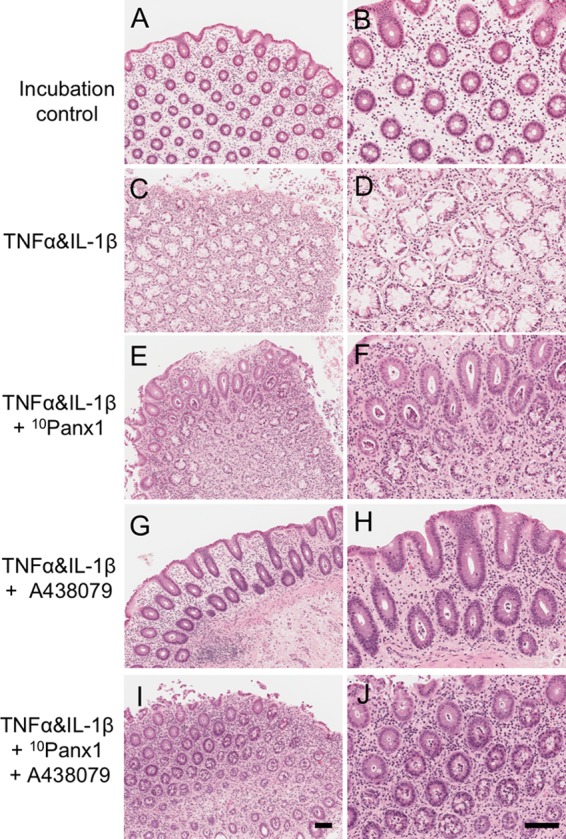
Histology of human colonic mucosa in the cytokine-induced colitis model. Hematoxylin and eosin (HE) was used to observe the histology of sections of human colonic mucosa (*n* = 9). Left column: 10×. Right column: 20×. **(A,B)** Incubation-control mucosa images showed intact crypt structure. **(C,D)** Incubation with cytokines, TNFα, and IL-1β (each 10 ng/mL), for 16 h caused mucosal destruction. **(E,F)** Co-incubation of the Panx1 channel blocker ^10^Panx1 (100 μM) reduced the amount of mucosal damage induced by cytokines. **(G,H)** Co-incubation of the P2X7R antagonist A438079 (100 μM) prevented cytokine induced mucosal damage. **(I,J)** The presence of both ^10^Panx1 and A438079 (both 100 μM) reduced some of the cytokine induced damage. Scale bars represent 100 μm. All images were taken from the colonic mucosa of a 50-year-old female patient.

Mucosal specimens suitable for histology following induction of colitis were chosen for all histological analyses (*n* = 9). Incubation-control mucosal strips showed the intact structure of the mucosal layer, particularly on the crypts and lumen (**Figures 2A,B**). Tissue incubated in cytokines alone (TNFα and IL-1β) showed either damaged or absent crypt structures (**Figures 2C,D**). The presence of either ^10^Panx1 or A438079 preserved the integrity of most mucosal structures, however, some damage was still apparent in some crypt structures (**Figures 2E–H**). Mucosal strips incubated with both inhibitors showed reduced cytokine-induced damage but to a lesser extent compared to the other drug treatment groups (**Figures 2I,J**).

**FIGURE 3:**
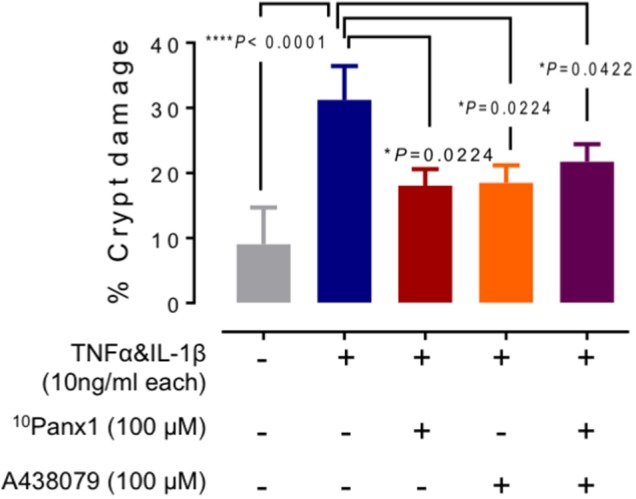
Analysis of mucosal damage in the cytokine-induced colitis model. Average area of structural damage from each tissue was measured and analyzed using one-way ANOVA followed by Holm–Sidak’s multiple comparison tests. Crypt damage in TNFα and IL-1β (each 10 ng/mL) treated mucosa was highly significant compared to the incubation-control group (*^∗∗∗∗^P* < 0.0001). Cytokines-induced damage was reduced in the presence of either ^10^Panx1 (*^∗^P* = 0.0224) or A438079 (*^∗^P* = 0.0224), and in the presence of both inhibitors (*^∗^P* = 0.0422).

Analysis of crypt damage showed a statistical significance between incubation-control and cytokine-incubated tissues (**Figure 3**, *P* < 0.0001). The presence of ^10^Panx1 showed a decrease in the percentage of crypt damage compared to the cytokine-only group (**Figure 3**, *P* < 0.05). Similarly, A438079 decreased the observed percentage of crypt damage when compared to the cytokine-only group (**Figure 3**, *P* < 0.05). In addition, a statistically significant decrease in crypt damage was observed in cytokine-induced crypt damage in the presence of both ^10^Panx1 and A438079 (**Figure 3**, *P* < 0.05).

#### ZO-1 Immunoreactivity

**FIGURE 4:**
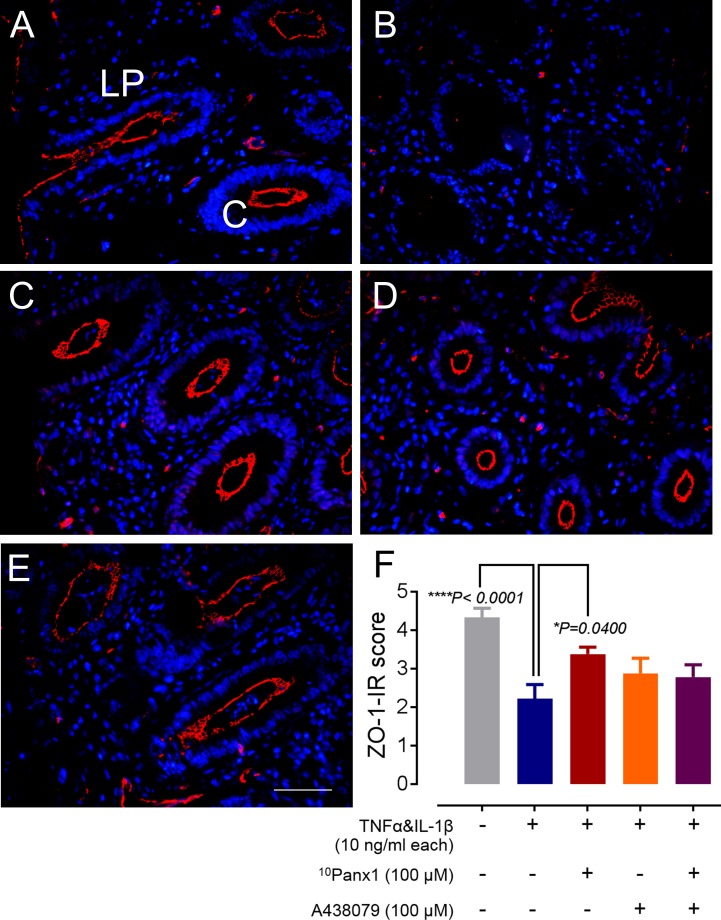
The immunoreactivity of tight junction protein ZO-1 (red) in mucosa colitis model. **(A)** Tight junctions in incubation-control tissues remained intact at both the crypts and lumen. **(B)** TNFα and IL-1β incubated mucosa tissues showed an absence of ZO-1-IR in the crypts and the lumenal cells. ZO-1-IR was present on most crypts and lumen structures in cytokine-incubated mucosa tissues with **(C)** ^10^Panx1, **(D)** A438079, and **(E)** both ^10^Panx1 and A438079. **(F)** Scores of ZO-1 probed sections were obtained using the criteria in **Table 1**. TNFα and IL-1β incubated mucosal tissues produced a statistically significant difference in scoring compared to incubation-control tissues, showing that cytokine-induced mucosa sections had a great reduction in ZO-1-IR (*^∗∗∗∗^P* < 0.0001). A statistical significance difference in scores was observed in ^10^Panx1 tissues co-incubated with cytokines when compared to the cytokine only group (*^∗^P* = 0.04), showing an increase in ZO-1-IR for the ^10^Panx1 treated group. Scale bar represents 50 μm. All images were taken from the colonic mucosa of a 50-year-old female patient.

ZO-1-IR was conducted to assess the tight junction integrity of each treatment group. Incubation-control tissue sections exhibited abundant ZO-1-IR showing intact tight junctions throughout most crypt and lumenal structures (**Figure 4A**). ZO-1-IR in cytokine-treated mucosal tissue was limited or absent in most specimens (**Figure 4B**). The presence of ZO-1-IR tight junctions in the drug-treated groups was varied in all specimens, however, there was a prevalence of ZO-1-IR in crypts compared to lumenal structures (**Figures 4C–E**). Scoring conducted on specimens from each treatment group showed statistical significance between the cytokine-only group and the incubation-control group (**Figure 4F**, *P* < 0.0001), emphasizing the absence of ZO-1-IR in cytokine-only incubation group. The presence of ^10^Panx1 with cytokines showed a decrease in ZO-1-IR scoring, implying that ^10^Panx1 partially prevented the loss of tight junctions caused by the presence of pro-inflammatory cytokines (**Figure 4F**, *P* < 0.05). Nevertheless, cytokines-induced increase of ZO-1-IR scoring was only marginally reduced in the presence of A438079 or both ^10^Panx1 and A438079 (**Figure 4F**).

#### Cell Counts of Leukocytes, Macrophages/Monocytes, and Mast Cells

**FIGURE 5:**
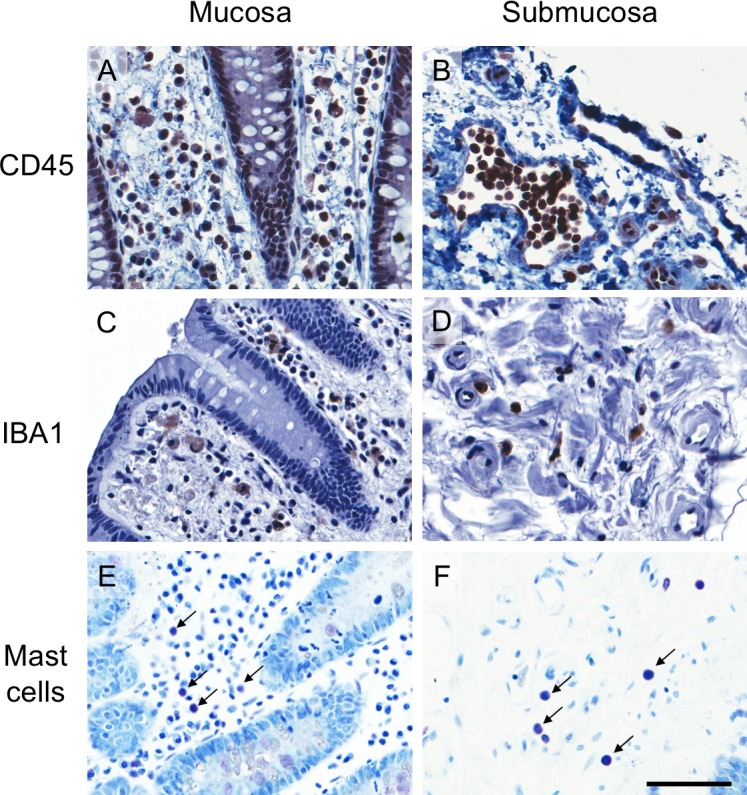
Images of CD45 immunoreactive (IR), IBA1-IR and toluidine blue stained cells from a representative incubation control tissue. **(A,B)** CD45-IR leukocytes, **(C,D)** IBA1-IR macrophages and monocytes, and **(E,F)** toluidine blue stained mast cells in mucosa (left panels) and submucosa (right panels). The scale bar represents 50 μm. All images were taken from the colonic mucosa of a 50-year-old female patient.

Cell counts of CD45-IR leukocytes, IBA1-IR macrophages and monocytes, and mast cells from toluidine blue staining were obtained from specimens for each treatment group. Leukocytes, macrophages/monocytes, and mast cells were found throughout the mucosal layer (**Figures 5A,C,E**) and submucosal layer (**Figures 5B,D,F**).

**FIGURE 6:**
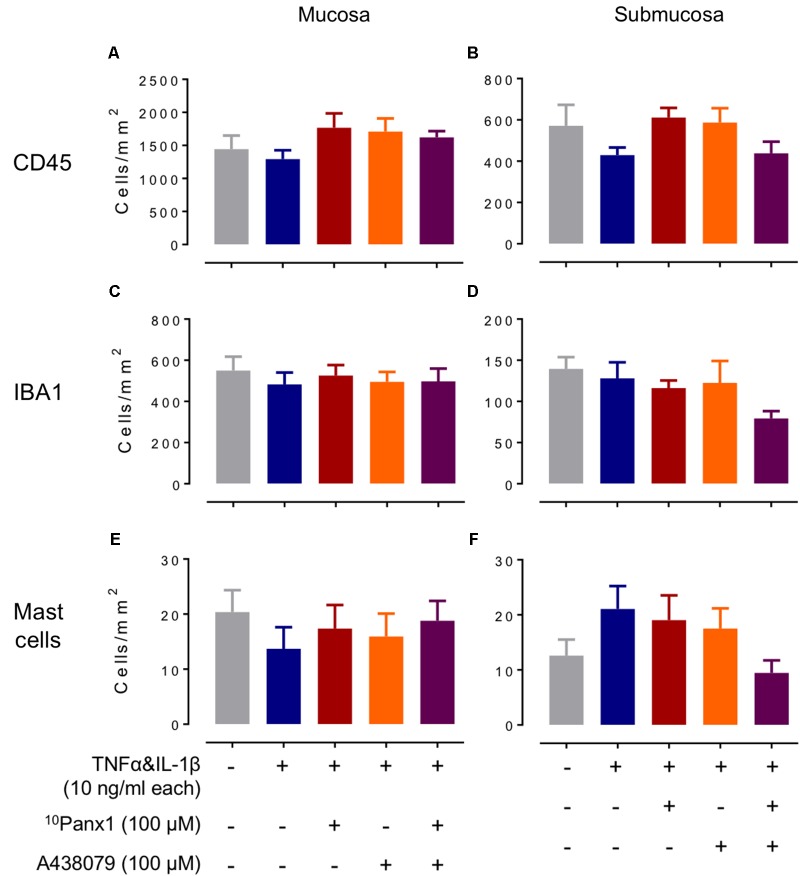
Cell counts of CD45-IR leukocytes, IBA1-IR macrophages and monocytes and toluidine blue stained mast cells in cytokines-induced colitis model. Cell counts of CD45-IR leukocytes **(A,B)**, IBA1-IR macrophages and monocytes **(C,D)**, and toluidine blue stained mast cells in mucosa **(E,F)** in mucosa (left panels) and submucosa (right panels). One-way ANOVA of cell counts did not reveal statistical significance between treatment groups in the mucosa, or in the submucosa (*n* = 9 specimens).

In the mucosa of the incubation-control group, positive CD45-IR cells were 1440 ± 210 cells/mm^2^ (*n* = 9). In the submucosal layer of the incubation-control group, CD45-IR leukocytes were less abundant than the mucosal layer, being 570 ± 100 cells/mm^2^ (*n* = 9). There were no significant differences between any groups in either mucosal or submucosal regions (**Figures 6A,B**).

The incubation-control group of the mucosal layer had a IBA1-IR cell count of 550 ± 70 cells/mm^2^ (*n* = 9), however, no statistical significance was found between treatment groups (*n* = 9, **Figure 6C**). In the submucosal layer of the incubation-control group, positive IBA1-IR cells were 140 ± 14 cells/mm^2^ which did not reveal any statistical significance when compared to other treatment groups (*n* = 9, **Figure 6D**).

No significant differences in mast cell counts were found between treatment groups in the mucosa, where the cell count in the incubation-control group was 20 ± 4 cells/mm^2^ (*n* = 9, **Figure 6E**, *n* = 9). Similarly, no significant differences in cell counts could be found in the submucosal layer between treatment groups, in which the cell count for the incubation group was 13 ± 3 cells/mm^2^ (*n* = 8, **Figure 6F**).

**FIGURE 7:**
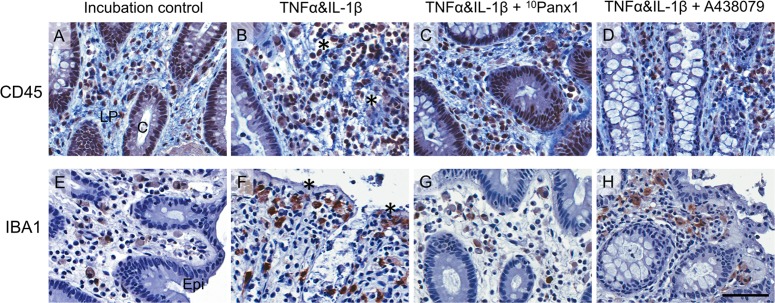
Images of CD45-IR leukocytes (top panels) and IBA1-IR macrophages and monocytes (bottom panels) in cytokines-induced colitis model from a representative human colon specimen. CD45-IR leukocytes **(A–D)** are densely and IBA1-IR macrophages **(E–H)** moderately localized in the lamina propria area. Note, in the TNFα and IL-1β treatment tissues, CD45-IR leukocytes migrated to the damaged crypts (indicated by ^∗^ in **B**) and more IBA1-IR macrophages were found aligning the damaged epithelium (indicated by ^∗^ in **F**). The scale bar represents 50 μm. C, crypt; Epi, epithelium; LP, lamina propria. All images were taken from the colonic mucosa of a 50-year-old female patient.

A migration of CD45-IR leukocytes to damaged crypts and IBA1-IR cells toward damaged lumenal sites was observed at varying degrees across all treatment groups (**Figures 7A–H**).

### Cytokine and Chemokine Levels of Bath Fluid From Cytokine-Induced Colitis Model

**FIGURE 8:**
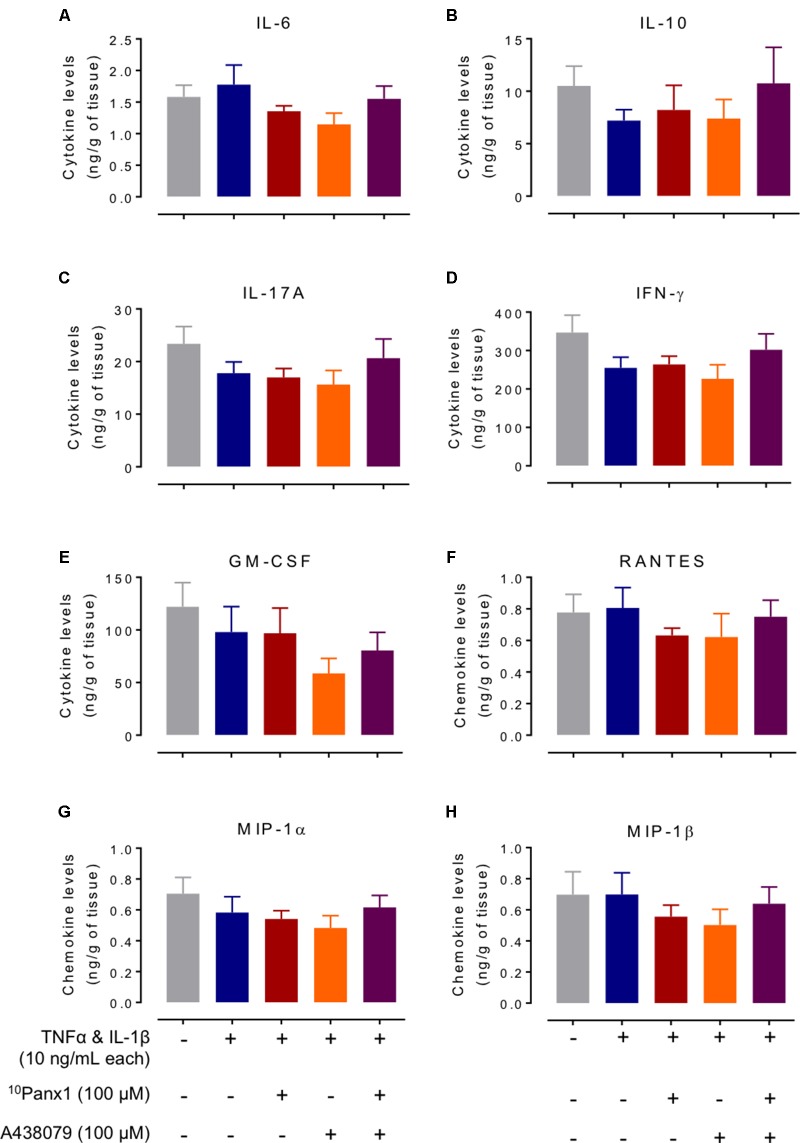
Levels of selected cytokines and chemokines from bath fluid of the colitis model. Cytokines and chemokines released from the tissues of the five treatment groups incubated over 16 h in bath fluid were measured: **(A)** IL-6, **(B)** IL-10, **(C)** IL-17A, **(D)** IFNγ, **(E)** GM-CSF, **(F)** RANTES, **(G)** MIP-1α, and **(H)** MIP-1β. One-way ANOVA analysis did not reveal any statistical significance between treatment groups.

Cytokines and chemokines that have been suspected to have roles in IBD were measured from bath fluid. A multiplex magnetic based kit was used to measure the bath fluid containing cytokines released from mucosal strips over the 16 h incubation period. The cytokine and chemokine levels from the incubation-control group (expressed as mean ± SEM ng/g tissue, *n* = 17–18) were: cytokines IL-6, 1.58 ± 0.19; IL-10, 10.5 ± 1.90; IL-17A, 21.2 ± 2.55; IFNγ, 347 ± 45.2 and GM-CSF 122 ± 23, and chemokines RANTES 0.78 ± 0.11, MIP-1α, 0.70 ± 0.11 and MIP-1β, 0.70 ± 0.15. ^10^Panx1 and A438079 on their own did not change the cytokine and chemokine levels compared to the control. A one-way ANOVA analysis did not show statistical significance for these cytokines and chemokines between different treatment groups (**Figure 8**).

### Epithelial Permeability of Cytokine-Treated Caco-2 Cells

**FIGURE 9:**
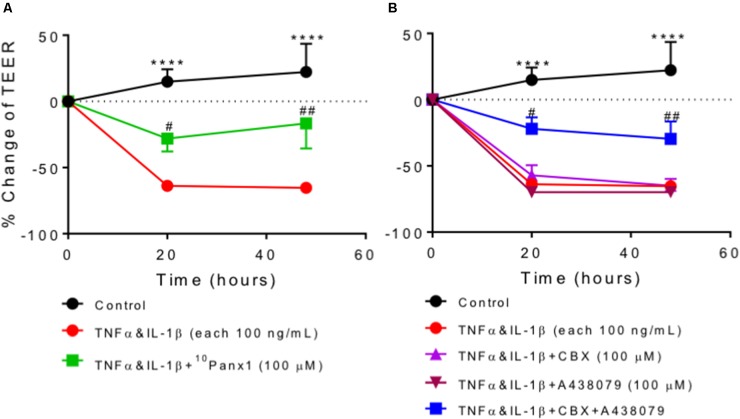
Transepithelial electrical resistance (TEER) in Caco-2 cells. Data were expressed as the percentage change of TEER relative to pre-treatment of the same well. Two-way ANOVA analysis followed by Holm–Sidak’s multiple comparison tests showed a highly significant reduction in TEER values after Caco-2 cells were incubated with cytokines TNFα and IL-1β (each 100 ng/mL) for 20 h and 48 h (^∗∗∗∗^*P* < 0.0001 compared to control). **(A)** Cytokines-induced TEER reduction was significantly prevented by co-incubation with ^10^Panx1 (100 μM). **(B)** The rise of cytokines-induced epithelial cell permeability was inhibited by the combined treatment with A438079 (100 μM) and the pannexin/connexin blocker CBX (100 μM), but A438079 and CBX on their own showed no protective effect. Each condition was measured as duplicates and repeated three to eight times.

The effects of Panx1 and P2X7R on epithelial permeability were examined in a human colonic epithelial cell line, Caco-2 cells, using the TEER method. Epithelial permeability was increased in Caco-2 cells incubated with TNFα and IL-1β (both 100 ng/mL), reflected by a marked reduction in TEER values, which was prevented by co-incubation with the Panx1 channel blocker ^10^Panx1 (**Figure 9A**). CBX, a pannexin/connexin channel blocker, and P2X7R antagonist A438079 alone failed to reverse TNFα and IL-1β-induced increase of cell permeability, but a combination of CBX and A438079 exerted a protective effect (**Figure 9B**).

## Discussion

In this study, using *ex vivo* mucosal explants of human colon we have shown that the normal structure of the colonic mucosa was severely disrupted by the incubation of mucosal strips with pro-inflammatory cytokines TNFα and IL-1β, resulting in crypt damage, loss of epithelial tight junction integrity and migration of lymphocytes and macrophages toward damaged sites. These results mirror the morphological features observed in the colon of IBD patients. More importantly, we have demonstrated that cytokines-induced mucosa damage was reduced in the presence of either the Panx1 channel blocker ^10^Panx1 and/or the P2X7R antagonist A438079. Furthermore, cytokines-treated Caco-2 cells also showed increased epithelial permeability which was attenuated in the presence of ^10^Panx1, and in the presence of both the pannexin/connexin channel blocker CBX and A438079.

Pro-inflammatory cytokines TNFα and IL-1β were used in the present study to induce a colitis-like state in the human colonic mucosa and Caco-2 cells. Consequently, significant crypt and epithelial cell damage was observed in the cytokines-only treated group compared to other experimental groups. This finding is consistent with the results from previous studies that have used these cytokines for the induction of colitis in the same tissues and cells (Harvey et al., 2013; Nicotra et al., 2013). Although we did not see an increased number of lymphocytes in the cytokines-treated group as reported in their studies (Harvey et al., 2013; Nicotra et al., 2013), we noticed a significant migration of CD45 positive lymphocytes to the damaged crypts and IBA1 positive macrophages toward the lumenal epithelium where epithelial cells fell off due to cell death. In addition, we have found that cytokines caused a marked reduction in ZO-1 immunoreactivity and increase in TEER values, indicating epithelial hyperpermeability and colonic barrier dysfunction resulting from inflammation-induced epithelial cell death.

We have found that the damage observed in the colitis model was significantly attenuated when the Panx1 channels were blocked by its inhibitor ^10^Panx1. Likewise, ZO-1 expression showed that colitis-induced disruption of epithelial tight junctions was significantly attenuated by ^10^Panx1, which was consistent with the results of Caco-2 cell permeability experiments. Panx1 has been previously shown to be involved in cell degradation during apoptosis. Panx1 was found to mediate the formation of apoptotic cell bodies during apoptosis which allows for normal disassembly of cells (Poon et al., 2014). However, in the presence of Panx1 inhibitors, disassembly is altered where instead abnormal cell membrane protrusions are produced, and smaller, disorganized apoptotic bodies are formed (Poon et al., 2014). The migration of leukocyte and microphage into damaged/injured tissue sites or toward chemotaxis has been well documented in both *in vivo and in vitro* studies, which involves complex mechanisms that are not year clear (for review, see Friedl and Weigelin, 2008; Pixley, 2012). A study by Qu et al. (2011) has found that liberating ATP and other unidentified “find me” signals through the Panx1 channels are essential for the recruitment of macrophages to apoptotic cells. It has been shown that the action of TNFα stimulating leukocyte migration through venous endothelial cells is via the activation of Panx1 channels (Lohman et al., 2015). In addition, Panx1 in conjunction with P2Y2R can mediate chemotaxis of neutrophils (Bao et al., 2013). These findings were consistent with the present observations that ^10^Panx1 attenuated inflammatory cytokines induced damage in crypts and CD45 and IBA1 positive cell migration. Our results may imply a regulatory role for Panx1 in cell death caused by inflammation in the human colon.

We and others have shown that Panx1 and P2X7R are densely expressed in the gastrointestinal tract (for review, see Diezmos et al., 2016). In the human colon, we have found that Panx1 is localized predominately on the glandular epithelium, leukocytes, blood vessel endothelium, nerve varicosities and erythrocytes within the mucosa (Diezmos et al., 2013), whereas, P2X7R immunoreactivity was found mainly on enteric neurons and some immune cells in the lamina propria of the mucosa which are suspected to be mast cells (data not shown). In addition, T cells have been shown to express Panx1 channels and P2X7R which are required for cell death (Shoji et al., 2014), and to form an ‘immune synapse’ that occurs during T cell activation and requires Panx1, P2X1R, and P2X4R (Woehrle et al., 2010a). Increasing evidence has shown that Panx1 plays a crucial role throughout the inflammatory response, from initiation through to resolution, and the action of Panx1 is closely tied to extracellular ATP signaling and purinergic receptor activities, especially those of P2X7R, since Panx1 is the key conduit of extracellular ATP release (Adamson and Leitinger, 2014). For instance, in the DSS mouse model of colitis, ATP released from CBX sensitive Panx1 channels activates P2X7R leading to inflammasome formation and NF-κB signaling (Wan et al., 2016). In the muscularis of the mouse colon, P2X7R stimulation of enteric neurons elicits a direct release of ATP onto enteric glia cells through the Panx1 channels, and ATP can in turn act on P2Y1R on enteric glia to increase intracellular calcium and elicit cell death in other enteric neurons (Gulbransen et al., 2012). Furthermore, overflow of ATP via P2X7R-activated Panx1 channels in reactive enteric glial cells affect neurotransmission in the myenteric plexus (Vieira et al., 2017). The exact role for Panx1 and P2X7R in inflammation and cell death is not clear and seems to vary depending on the experimental models. Although CBX is an inhibitor for both Panx1 and connexin hemichannels, its effect on the Caco-2 cells is less likely via the blockage of connexin hemichannels since these channels are closed at the normal Ca^2+^ range (Fasciani et al., 2013).

Paracrine signaling involving an increase in ATP release may contribute to the damage in the crypts and tight junctions seen in this study. Since severe tissue cell damage was seen in cytokines-treated group, we expected to see a substantial amount of ATP released into the tissue milieu from multiple cells including immune cells and injured/dead cells as shown in the mouse colon after feeding with 4% DSS for 5 days (Wan et al., 2016). Surprisingly, the amount of ATP released from human colonic mucosal strips into the culture media was similar between groups, from 9.79 to 13.15 pmol/mg tissue weight (Supplementary Figure 4). In their study, ATP liberated from the mouse colon was measured 1 h after the animals were sacrificed (Wan et al., 2016). In contrast, we measured the amount of ATP in culture media after mucosal strips were incubated for 16 h. Colonic mucosal tissue is rich in membrane-bound ectonucleotidases and the ecto-5′-nucleotidase, hydrolyzing extracellular ATP to ADP, AMP and adenosine (Lavoie et al., 2011), therefore, we cannot rule out the possibility that tissue damage enhancing extracellular ATP had occurred but the changes in ATP levels between groups were masked by ATP degradation after a prolonged incubation period. Although it is ideal to measure ATP released from Panx1, the ATP from damaged cells need to be taken into account and can be a huge confounder of the results, particularly after 16 h of incubation. Since cytokines-induced colonic mucosa damage was partially inhibited but not abolished by the blockage of Panx1 channel and/or P2X7R, other pathways for cell death independent of Panx1 and/or P2X7R may have also been activated by exogenous TNFα and IL-1β, for example, autophagy or necrosis (Bialik et al., 2010). Indeed, Panx1 channels and P2X7R can act independently of each other in enhancing multinucleated macrophage formation (Crespo Yanguas et al., 2017); some cells do not require Panx1 function to elicit a specific effect, such as in murine macrophages and in mast cells (Kurashima et al., 2012; Alberto et al., 2013).

It has been suggested that dysregulation of tight junction proteins is involved and may precede the development of IBD (Landy et al., 2016). In a previous study of ulcerative colitis and Crohn’s disease biopsies, ZO-1 expression was reduced along with an up-regulation of another tight junction protein claudin-2 (Das et al., 2012). In a study of experimental colitis in mice, anti-TNF drugs, such as infliximab and etanercept, attenuated inflammation induced reductions in ZO-1 and occludin (Fries et al., 2008). Interestingly, TNFα has previously been shown to increase tight junction permeability via the activation of myosin light chain kinase promoter which leads to tight junction barrier opening (Ma et al., 2004; Ye et al., 2006). However, tight junction disruption does not always lead to colitis (Khounlotham et al., 2012). This suggests that although a compromised epithelial barrier is a characteristic of IBD, the loss of tight junctions does not necessarily lead to the development of other colitis symptoms.

Although our results suggest that the blockage of Panx1 could effectively protect colonic epithelial cells from inflammatory mediators-induced cell damage, the role of P2X7R in this aspect is less clear. The P2X7R antagonist A438079 significantly reduced cytokines induced crypt damage, but its effect on ZO-1 expression was marginal, which was consistent with the results from the Caco-2 cell permeability experiments. P2X7R is well documented in relation to its role in the development and progression of inflammation. It has been shown that P2X7R activation induces the release of IL-1β and TNFα and other pro-inflammatory cytokines from macrophages and microglia (Hide et al., 2000; Lister et al., 2007; Monif et al., 2016). The action of P2X7R appears to be upstream of cytokine release, which may explain why the blockage of P2X7R by A438079 is less effective to the exogenous application of TNFα and IL-1β. Interestingly, studies have shown that high doses of ATP induce rapid release of proinflammatory mediators, but chronic exposure to low-dose ATP activates dendritic cells and macrophages to secrete anti-inflammatory cytokines IL-10 and IL-1 receptor antagonist IL-1RA, suppressing inflammation (la Sala et al., 2003; Lister et al., 2007). These observations suggest that purinergic signaling may mediate the interplay between pro- and anti-inflammatory mechanisms.

The levels of cytokine and chemokine released into media were similar in different experimental groups in the present study, despite observing changes to the mucosal structure and immune cell migration. It may be possible that other unmeasured cytokines involved with Panx1 and P2X7R were altered such as IL-2 and IL-8 (Woehrle et al., 2010a,b; Wei et al., 2016). Nevertheless, the present study showed similar results to a previous study which used a similar colitis model, where IL-17A levels were not influenced by the presence of TNFα and IL-1β (Harvey et al., 2014). Furthermore, cytokines and chemokines in the present study may have experienced a transient increase during the 16 h incubation. In a previous study, hemokinin-1, a pro-inflammatory mediator of the tachykinin family, stimulated the release of cytokines and chemokines such as MIP-1α, MIP-1β and RANTES from human colonic mucosa after a 4 h incubation period (Dai et al., 2011). Thus, the cytokines and chemokines in the present study may have been degraded after their release due to the longer 16 h period of incubation. The lack of changes in cytokine levels in the present study may also be associated with the lack of changes in immune cell numbers, which is likely the result of absence of blood flow in an *ex vivo* colitis model such as in the present study. Nevertheless, a determination of cytokine and chemokine protein expression in colonic tissue homogenates may offer useful information about if the levels of cytokines and chemokines produced are varied between groups.

While the role of Panx1 and P2X7R in modulating inflammatory and immune cell activities is well established, their impact on epithelial cell damage during inflammation is less clear. In the present study, exogenously applying TNFα and IL-1β caused severe crypt and epithelial cell damage, which was significantly lessened by the application of the Panx1 channel blocker ^10^Panx1 and the P2X7R antagonist A438079. Although the exploration of the underlying mechanism was beyond the scope of the current study, it is reasonable to assume that cytokines-induced cell death caused a substantial increase in extracellular ATP, which in turns triggered P2X7- and Panx1-dependent cell apoptosis, further damaging tissues. It is worthwhile noting that in recent years, increasing evidence has revealed the great importance of epithelial cells and their barrier function in the development of chronic inflammation in IBD. Promoting mucosal healing and maintaining long-term remission have become the therapeutic standard for IBD (Okamoto and Watanabe, 2016).

In summary, one of the advantages of this study was the use of native intact human colonic mucosal tissue to avoid marked species differences in inflammatory and tissue repair responses and in the responses to drugs. Furthermore, the *ex vivo* colitis model used in this study closely resembled the morphological and histopathological features of human IBD. An important and original finding of this study is that the blockade of Panx1 channels and P2X7R reduced the inflammatory cytokines-induced crypt damage, loss of tight junctions and increase in cell permeability, thus suppressing colonic inflammation. The study supports the theory that Panx1 and P2X7R have roles in mediating inflammation and cell death, and the likely mechanism of action is via the activation of the Panx1 channels and P2X7R from the multitude of cell types in the colonic mucosal layer. This is particularly evident for Panx1 and P2X7R on colonic epithelial cells, as demonstrated in the present study by the fact that inflammatory cells cannot originate from blood supply in this model, the migration of parietal inflammatory cells toward the damaged epithelium, and the lack of changes in the level of major cytokines and chemokines. Therefore, Panx1 and P2X7R on epithelial cells may be potential therapeutic targets for the treatment of IBD.

## Author Contributions

ED: performed the research, analyzed the data, and wrote the manuscript. IM: performed the cell culture experiments. LZ, SS, and PB: contributed to the writing of the manuscript and essential reagents. DP and SG: provided the human specimens and patient information. LL: designed the research study, contributed to the writing of the manuscript, and provided the essential reagents and tools.

## Conflict of Interest Statement

The authors declare that the research was conducted in the absence of any commercial or financial relationships that could be construed as a potential conflict of interest.

## Abbreviations

DSS: dextran sulfate sodium
FCS: fetal calf serum
GM-CSF: granulocyte-macrophage colony-stimulating factor
HE: hematoxylin and eosin
IBD: inflammatory bowel disease
MIP: macrophage inflammatory protein-1
Panx1: pannexin-1
P2X7R: P2X7 receptors
RANTES: regulated on activation, normal T cell expressed and secreted
ZO-1: zonula occludens-1
