# Supplementary material for: Blockade of Pannexin-1 Channels and Purinergic P2X7 Receptors Shows Protective Effects Against Cytokines-Induced Colitis of Human Colonic Mucosa
Source: Front Pharmacol. 2018 Aug 6;9:865. doi: 10.3389/fphar.2018.00865 (PMC6087744; doi:10.3389/fphar.2018.00865)
Supplement: Supplementary file 1 [file Table_1.PDF]

Supplementary Table 1. Primary and secondary antibodies for immunohistochemistry studies.

Antibody	Host	Dilution	Source
Anti-CD45 antibody	Rabbit	1:2000	Abcam ab10558
Anti-IBA1 antibody	Rabbit	1:2000	Wako 019-19741
Anti-ZO-1 Antibody	Rabbit	1:200	Invitrogen 61-7300
Anti-Rabbit IgG H&L (Alexa Fluor® 568) preadsorbed	Donkey	1:200	Abcam ab175692
Biotinylated Anti-Rabbit IgG Antibody	Goat	1:200	Vector BA-1000
