# Supplementary material for: Blockade of Pannexin-1 Channels and Purinergic P2X7 Receptors Shows Protective Effects Against Cytokines-Induced Colitis of Human Colonic Mucosa
Source: Front Pharmacol. 2018 Aug 6;9:865. doi: 10.3389/fphar.2018.00865 (PMC6087744; doi:10.3389/fphar.2018.00865)
Supplement: Supplementary file 2 [file Image_1.PDF]

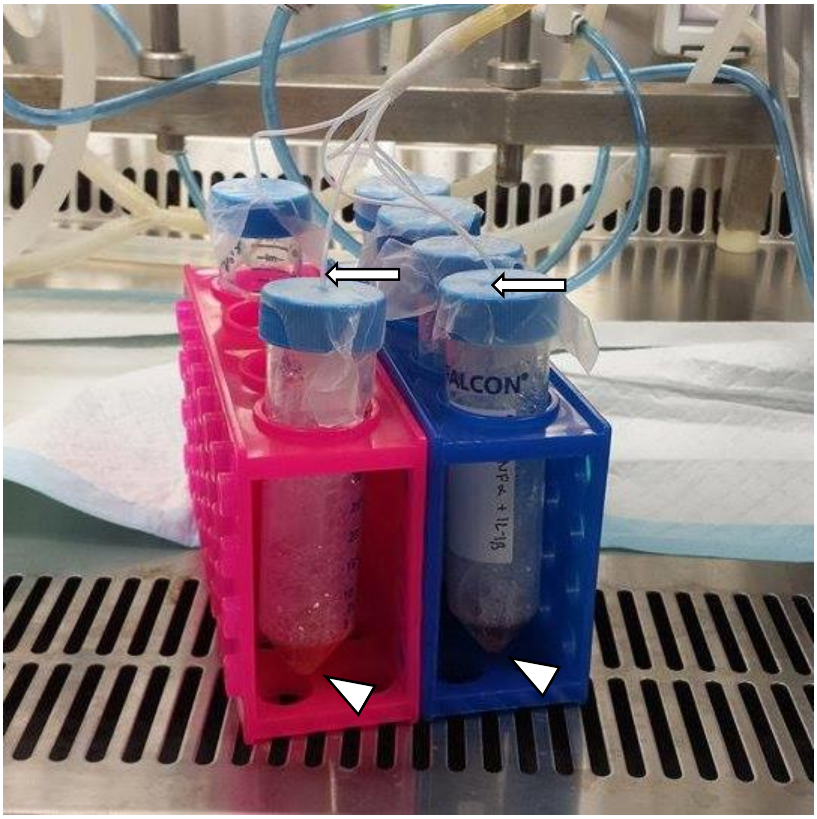

Supp Figure 1. Equipment setup for human colonic mucosa colitis model. Conical centrifuge tubes (50 mL) were set up in plastic racks. Tubings for carbogen were fed through a hole in the lid of conical centrifuge tubes (arrows) and held in place using parafilm. Each tube contained 3 mL of RPMI 1640 media with 1% FCS and 1% penicillin-streptomycin, and 2 mucosal strips (arrow heads). The setup was placed in a 37^{0}C water bath (not shown) for 16 h, the optimised incubation time (see Supp Figure 3).
