# Supplementary material for: Blockade of Pannexin-1 Channels and Purinergic P2X7 Receptors Shows Protective Effects Against Cytokines-Induced Colitis of Human Colonic Mucosa
Source: Front Pharmacol. 2018 Aug 6;9:865. doi: 10.3389/fphar.2018.00865 (PMC6087744; doi:10.3389/fphar.2018.00865)
Supplement: Supplementary file 3 [file Image_2.pdf]

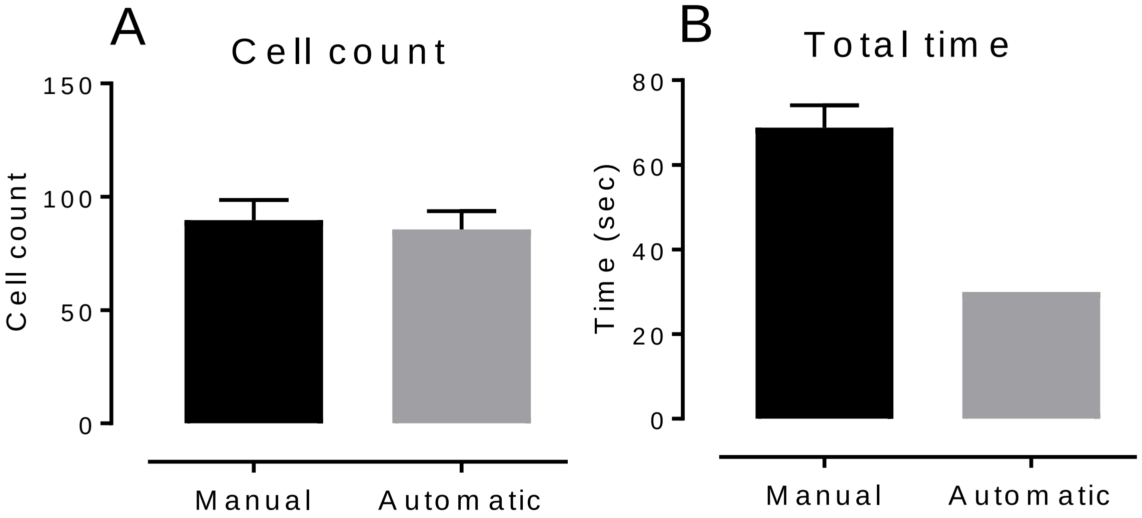

Supp Figure 2. Cell counting using the ‘analyse particles’ command from ImageJ. Six representative images from CD45 cell counts were processed for both manual and automatic cell counting, the latter using the ‘analyse particles’ command. (**A**) Cell counts of manual (mean; 90 cells) and automatic (mean; 86 cells) showed similar results. (**B**) Total time for automatic method (mean; 30 sec) showed more efficiency in obtaining results compared to manual cell counting (mean; 69 sec).
