# Supplementary material for: Blockade of Pannexin-1 Channels and Purinergic P2X7 Receptors Shows Protective Effects Against Cytokines-Induced Colitis of Human Colonic Mucosa
Source: Front Pharmacol. 2018 Aug 6;9:865. doi: 10.3389/fphar.2018.00865 (PMC6087744; doi:10.3389/fphar.2018.00865)
Supplement: Supplementary file 4 [file Image_3.PDF]

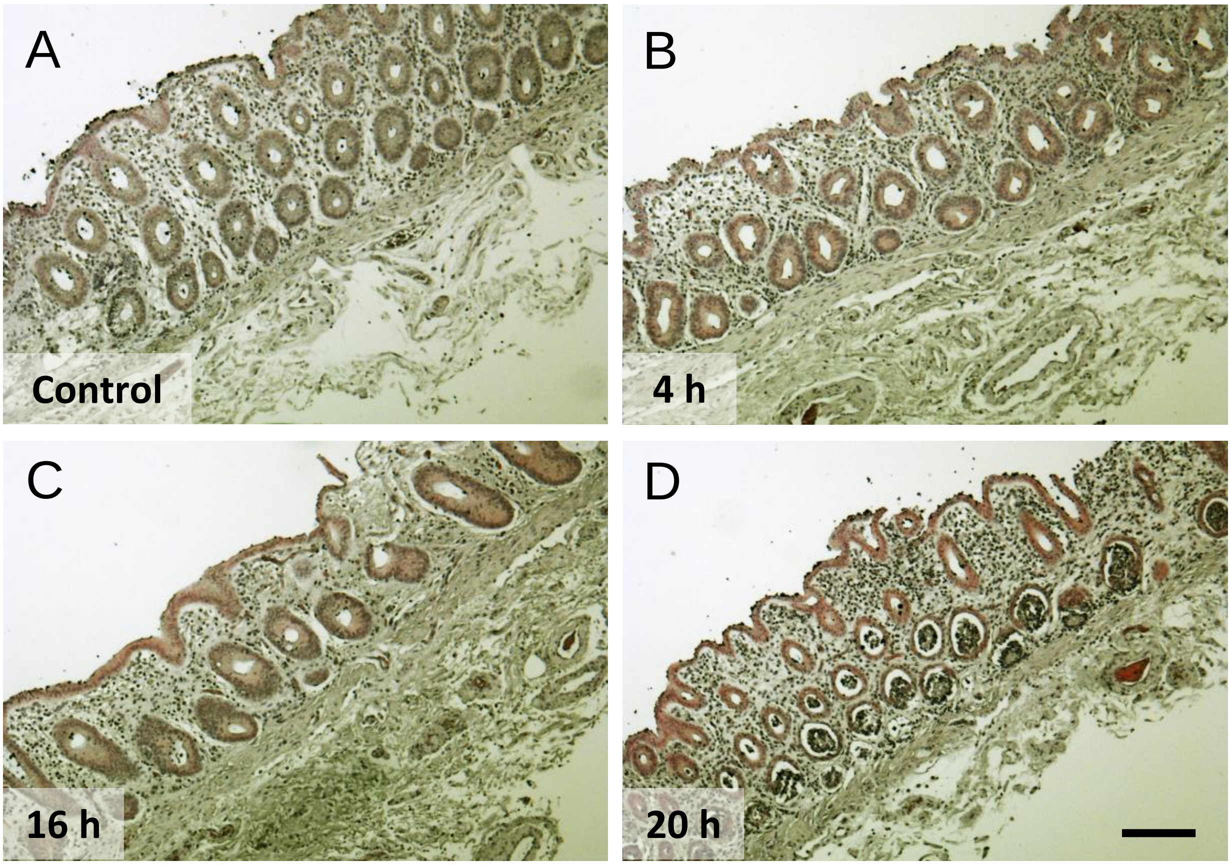

Supp Figure 3. Optimisation of incubation time for cytokines-induced colitis model. Mucosal strips (4 × 10 mm) were placed in conical centrifuge tubes set up as depicted in Supp Figure 1. Mucosal strips were subjected to 4 h, 16 h, and 20 h incubation times, sectioned and stained (H&E) to determine the longest time period tissue integrity could be maintained. (**A**) Control (no incubation time, fixed immediately in Zamboni) mucosa showed the integrity of the lumenal epithelial cells and crypt structures maintained. (**B**) Mucosal structures after 4 h incubation time were maintained. (**C**) Similarly, mucosal structures after the 16 h incubation time were maintained. (**D**) After 20 h, some mucosal damage at the crypts closest to the muscularis mucosae could be observed. Scale bar represents 100 μm.
