# Supplementary material for: Blockade of Pannexin-1 Channels and Purinergic P2X7 Receptors Shows Protective Effects Against Cytokines-Induced Colitis of Human Colonic Mucosa
Source: Front Pharmacol. 2018 Aug 6;9:865. doi: 10.3389/fphar.2018.00865 (PMC6087744; doi:10.3389/fphar.2018.00865)
Supplement: Supplementary file 5 [file Image_4.PDF]

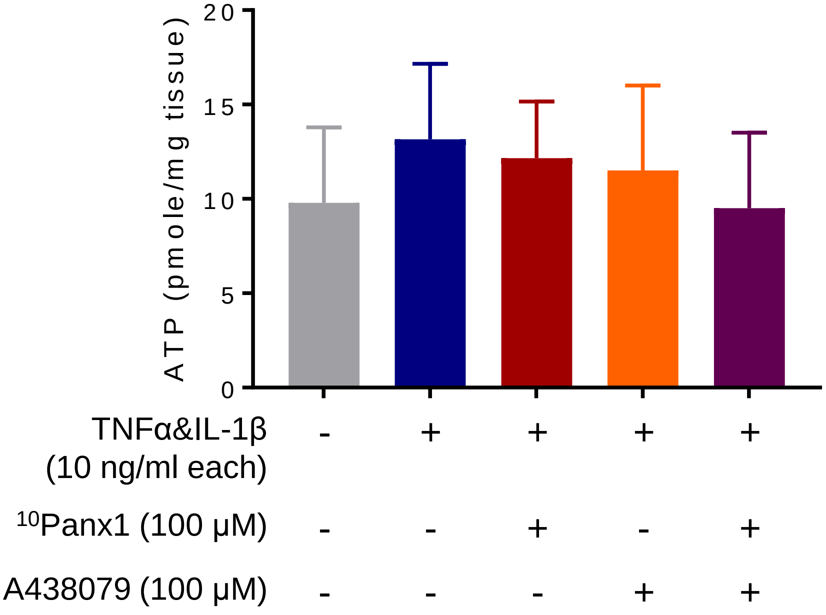

Supp Figure 4. Level of ATP from bath fluid of the colitis model. ATP released from the colonic mucosal tissues of the five treatment groups incubated over 16 h in bath fluid were measured by using the ATP Bioluminescence method. One-way ANOVA analysis did not reveal statistical significance between treatment groups.
